# Neurological, cognitive and learning evaluation of students who were born preterm

**DOI:** 10.1590/1984-0462/2022/40/2020252

**Published:** 2021-07-30

**Authors:** André Luis Santos do Carmo, Fernanda Wagner Fredo, Isac Bruck, Joseli do Rocio Maito de Lima, Rebecca Nóbrega Ribas Gusso Harder Janke, Thais da Glória Messias Fogaça, Jacqueline Andrea Glaser, Tatiana Izabele Jaworski de Sá Riechi, Sergio Antonio Antoniuk

**Affiliations:** a Universidade Federal do Paraná. Curitiba, PR, Brasil.

**Keywords:** Infant, premature, Learning disabilities, Developmental disabilities, Recém-nascido prematuro, Deficiências da aprendizagem, Deficiências do desenvolvimento

## Abstract

**Objective:**

To evaluate the cognitive and academic profile of preterm newborns at school age and to determine the factors related to prematurity and sociodemographic profile that influence these results.

**Methods:**

Patients aged 6-14 years old that were assisted in the preterm follow-up clinic were recruited. The cognitive, academic, and neurological capacities were accessed through a detailed evaluation with a child neurologist, a neuropsychologist and a psychopedagogue. Neonatal data were collected from patient records.

**Results:**

97 children were included and 14 were excluded from the study, resulting in 83 children. Gestational age (GA) was 30±3 weeks and weight at birth was 1138g (605 to 4185g). Poor performance was shown in 38.4% for writing, 57.5% for reading and 42.5% for mathematics. The mean total intelligence quotient (IQ) was 96±14.9 points, and 10.9% were considered altered. Children with unstructured families presented 78.3% of failure in reading tests (p=0.029). The multivariate analysis showed association between GA at birth and classic mini-mental score (p=0.043), total IQ (p=0.047), perceptual organization IQ (p=0.035), and processing speed IQ (p=0.036). There was also association between weight at birth and the classic (p=0.004) and adapted (p=0.007) mini-mental scores; invasive mechanic ventilation duration and classic mini-mental (p=0.049); and lower maternal age and processing speed IQ (p=0.033).

**Conclusions:**

Preterm infants at school age had high frequency of failure in cognitive and academic evaluation tests. Learning difficulties are high among them. Multiple neonatal variables are related with altered cognitive and students development.

## RESUMO

**Objetivo:**

Avaliar o perfil de desenvolvimento cognitivo e acadêmico de recém-nascidos pré-termo em idade escolar e indicar os fatores relacionados à prematuridade e ao perfil sociodemográfico que influenciam esse resultado.

**Métodos:**

Recrutaram-se pacientes com idades entre 6 e 14 anos que fizeram seguimento no ambulatório de acompanhamento de prematuros. As capacidades cognitiva, acadêmica e neurológica foram acessadas por avaliação pormenorizada com neuropediatra, neuropsicóloga e psicopedagoga. Buscaram-se os dados neonatais nos prontuários.

**Resultados:**

Foram incluídos 97 recém-nascidos pré-termo e excluídos 14, resultando em 83 crianças. A idade gestacional foi 30±3 semanas e o peso de nascimento 1138g (605; 4185g). Tiveram mau desempenho em escrita 38,4%, leitura 57,5% e matemática 42,5%. A média de quociente de inteligência total foi de 96,0±14,9 pontos, sendo 10,9% considerados alterados. Crianças com famílias desestruturadas apresentaram 78,3% de falha de leitura (p=0,029). Pela regressão multivariada, houve relação entre idade gestacional ao nascimento e pontuação no miniexame do estado mental (minimental) clássico (p=0,043), quociente de inteligência total (p=0,047), quociente de inteligência organização perceptual (p=0,035) e quociente de inteligência velocidade de processamento (p=0,036); entre peso ao nascer e minimental clássico (p=0,004) e adaptado (p=0,007); entre tempo de ventilação mecânica invasiva e minimental clássico (p=0,049); e entre idade materna mais baixa e quociente de inteligência velocidade de processamento (p=0,033).

**Conclusões:**

Os recém-nascidos pré-termo apresentaram alta frequência de falha nos testes de avaliação cognitiva e acadêmica. Uma série de intercorrências neonatais apresenta associação com alterações no desenvolvimento cognitivo e escolar.

## INTRODUCTION

Despite the therapeutic advances in the past decades regarding premature infant (PI) care, the rates of long-term sequelae have remained high.^1^ About 10 to 15% of PIs who survive the neonatal period present with severe neurological or sensory sequelae, such as severe intellectual disability, tetra spastic cerebral palsy (CP) or blindness, and require support for the development of activities of daily living throughout life.^2^ Of the others, 30 to 60% will present with minor difficulties, such as mild intellectual disability, delayed language development and emotional and behavioral issues.^1^

Considering extreme premature infants (EPI), that is, those born before the 28^th^ week of gestational age (GA), the level of severe sequelae can be even higher, reaching 32%, including intellectual disability (25%), CP (11%) and autism (7%), presented isolated or in multiple associations.^3^

The factors that have the most negative impact on development and increase the risk of delay and disabilities, especially in terms of cognition, are low GA and low weight. Other perinatal factors that increase the risk of developing severe intellectual disabilities are severe peri-intraventricular hemorrhage (PIVH), cystic periventricular leukomalacia, use of corticoids after birth and going through major surgical procedures.^4^ Factors such as being male, ethnicity (non-white), low income and low parental schooling are also an influence.^5^ The following can be listed as protective factors: breastfeeding, higher family income, insertion in programs of early stimulation and preventive educational support.^6^ ^,^ ^7^

Considering the high risk of delayed development, a series of methods and screening tests was created and implemented in PI follow-up outpatient clinics all over the world, with high levels of success and social return. However, up until this moment it is not possible to accurately determine, using traditional scales and developmental tests, which PIs will present with school-related difficulties.^8^ ^,^ ^9^ ^,^ ^10^

One of the reasons indicated for this difficulty lies on the fact that learning (especially reading, writing and mathematics) is a determined complex cognitive function, modified and modulated not only by biological factors, but also environmental, social and cultural aspects.^11^ ^,^ ^12^ Methodological limitations make it harder to assess and understand the complex social skills of PIs when they reach school age.^13^

PIs have increased risk of developing Attention Deficit Hyperactivity Disorder (ADHD), whose prevalence is around 20 to 25% in this population, and the severity of symptoms is inversely proportional to weight at birth and GA.^8^ ^,^ ^14^ Likewise, the frequency of intellectual disability (ID) among PIs reaches levels as high as 25 to 41%, being higher among EPIs.^3^ ^,^ ^15^ ^,^ ^16^ When compared with children born at term, PIs at any GA present 11 to 15 fewer points of intelligence quotient (IQ) than children born at term.^17^ ^,^ ^18^ Among PIs, those who grow up in an unfavorable social environment present higher chances of developing intellectual disability.^19^ Also, PIs present higher prevalence rates^20^ of difficulties in reading (22%), writing (20%) and mathematics (40%), and about 2.8 more chances of requiring special educational assistance than their peers born at term.^21^

The objective of this study was to make a survey of the national profile of intellectual disability and school-related difficulties among PIs followed-up at an outpatient clinic for children with developmental risks, as well as to identify which factors related to prematurity and sociodemographic profile may be related to these outcomes, due to the lack of data regarding long-term cognitive development in PIs who survived without severe sequelae.

## METHOD

This is an ambispective, cross-sectional, analytical and observational study. It was conducted between November, 2015, and September, 2019. The evaluation of patients and data collection occurred between November 2015 and April 2019.

Inclusion criteria were: parents or tutors signing the informed consent form; patients signing the assent form, when applicable; GA equal to or inferior than 36 weeks at birth, according to the data registered in the records (considering the calculation of GA ultrasound performed until the 12^th^ week of GA and chronological and menstrual data); having been born between 2003 and 2012; having attended at least three appointments with the child neurologist. Exclusion criteria were: patients who presented with clear ID, autism or another neurodevelopmental disorder after the initial medical assessment, according to the clinical criteria established in the Diagnostic and Statistical Manual of Mental Disorders^22^; patients enrolled in special schools.

The database of the outpatient clinic was used to contact the parents of the patients who met the inclusion and exclusion criteria, using telephone numbers from the hospital’s records, in order to invite them to participate in the study.

The verification of the neuropsychomotor development of children at school age was performed by clinical examination and tests conducted by the child neurologist. The tests were the pediatric symptom checklist (simple instrument, fast to apply, in which parents or tutors mark the presence of symptoms regarding the mental health of the child and grade them between very present or medium present);^23^ the Vanderbilt assessment scale for lack of attention and hyperactivity, to be filled out by the parents (composed of objective questions about the child’s behavior, grading the symptoms in four categories for each item: never, occasionally, often and very often, being considered altered when the two last categories are marked. There are nine items for lack of attention and nine for hyperactivity. The questionnaire is considered altered when more than six items are marked for each);^24^ the classic mini-mental state examination (cognitive screening test that assesses orientation, immediate memory, attention and calculation, remembrance, language and visuoconstructive praxis), adapted mini-mental for the pediatric age group (assessing orientation, attention, concentration, sensory perception, immediate memory, language and visuoconstructive praxis)^25^ ^,^ ^26^ and the evolutionary neurological examination (a pediatric neurological assessment of abilities and aspects of childhood development used to classify the development of children aged from 3 to 7 years in different domains. It includes the analysis of static balance, dynamic balance, appendicular coordination, trunk-limb coordination, motor persistence and gnosis. Each of these items includes expected activities for a specific age, and the result is considered altered when the child does not reach them).^27^ In the analysis, to define the test results as altered, we considered 28 as the cut off point for the symptom checklist^28^, 20 points in children aged 7 years, and 28 in children aged more than 8 years for the classic mini-mental^26^ ^,^ ^29^ and for the adapted mini-mental,^26^ ^,^ ^29^ respectively.

The educational analysis was carried out by a psychopedagogue who used reading, writing and mathematical tests, both original and standardized, created by the staff and adapted for each age group and academic grading. Cultural, local and regional characteristics were considered in order to assess performance in the fields of reading, writing and mathematics. After correction, according to the number of errors, the patients were classified as sufficient or insufficient in that skill, considering the age group and the school year,^30^ as well as through the Piaget operational stage (which assesses the reasoning and provides the cognitive potential of the child according to each age group. It is considered altered when the child is below the expectation for the age group).^30^ ^,^ ^31^ The academic tests evaluated the performance of children in school abilities according to grade and age. The IQ test was executed by a psychologist using the Wechsler Intelligence Scale for Children - 4^th^ edition (WISC-4), divided in verbal comprehension, perceptual reasoning, attention skills and processing speed^32^. It was considered as altered when lower than 80.

The analyzed variables were: sex, maternal age at birth, gestational age at birth, maternal schooling, family income, family structure (the family was considered unstructured at the absence of at least one parent), maternal serology, weight at birth, Apgar score at 1 and 5 minutes, presence of early and late neonatal sepsis, days of mechanic ventilation, days of hospitalization in the neonatal ICU, presence of retinopathy of prematurity, presence of neonatal jaundice, need for blood transfusion for volemia replacement, need for blood transfusion, presence of HPIV, performance in the classic and adapted mini-mental tests, evolutionary neurological examination, symptom checklist, result of the Vanderbilt Assessment Scale, presumptive clinical diagnosis, evaluation of the Piaget operational stage, performance in reading, writing and mathematics, and IQ results.

Data from the perinatal period were obtained through records in the charts referring to that time, and socioeconomic data were collected during the child neurologist appointment. The evaluations of the multiprofessional staff (medical, pedagogical and psychological) were not made on the same day to prevent patients’ fatigue; there was an interval of up to 14 days between them.

The data were clustered in Excel^®^ spreadsheets and analyzed with Statistica^®^, using simple descriptive analysis, Fisher’s Exact test and Pearson’s chi-squared test for categorical variables. Multiple regression was performed with the following independent variables: GA at birth, weight at birth, Apgar score at 1 and 5 minutes after birth, days of hospitalization, days of invasive mechanic ventilation, days of non-invasive mechanic ventilation, maternal age and family income. The analyzed dependent variables were: score in the symptom checklist, score in the classic and adapted mini-mental, total IQ, verbal comprehension IQ, perceptual reasoning IQ, processing speed IQ and operational memory IQ

The study was approved by the Research Ethics Committee and registered with number 57932616.2.0000.0096.

## RESULTS

**Figure 1.**
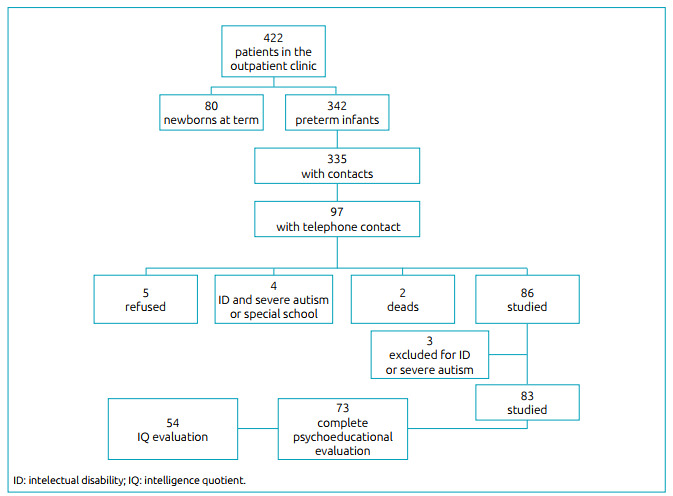
Recruitment of participants.

We verified the records of 442 patients in the book maintained in the outpatient clinic, all of whom were born from 2003 and 2013. Of these, 342 patients were premature, being the GA at birth equal to or inferior than 36 weeks. Of these, we obtained the telephone number of 335 in the hospital records. We could contact 97 patients (whose number was updated in the hospital database), and 5 did not accept the invitation to participate in the study; 2 died due to other intercurrences; and 4 were excluded for not matching the criteria. So, 86 children were assessed, of whom 2 were identified with severe ID in the first medical assessment and could not complete the evaluations; 1 had severe autism, among other intercurrences, and died during the study. Finally, we assessed 83 children (Figure 1).

Because the evaluations of each professional were not made on the same visit, in order to prevent fatigue among participants, the absence rate was high, as well as loss of follow-up. So, 83 children were submitted to medical evaluation, only 71 parents answered the Vanderbilt Assessment Scale; 73 children underwent psychoeducational evaluation; and 54 children underwent the IQ tests.

Of the analyzed patients, 60.2% were boys, and 39.8% were girls. According to family income, median was R$ 1,850,00 (300-5,000); 47.7% had family income lower than 2 minimum wages. When assessing family structure, 24.1% lived only with their mother or grandmothers. As to maternal schooling, 16.6% had completed elementary school (up to eight years of formal schooling). Other data regarding demographic profile, perinatal history and results of the evaluation at the school age can be found in Table 1.

**Table 1:** Demographic profile, perinatal history and results of the evaluation at school age.

	Mean
Age in years	8.6±1.7
School year	3.0±1.5
Maternal age at birth in years	28.0±7.1
GA at birth in weeks	30.0±3.5
Weigh at birth in grams	1354.0±623.5
Weight at hospital discharge in grams	2098.0±419.8
Apgar Score at 1 minute	5.0±2.4
Apgar Score at 5 minutes	8.0±1.6
Days of hospitalization	49.0±30
Days with invasive mechanic ventilation	5.0±9.3
Days in CPAP	5.0±8.8
Family income in reais	2286.0±1213.5
Number of siblings	1.0±1.2
Symptom checklist	17.0± 9.3
Score in classic mini-mental	22.0±7.2
Score in adapted mini-mental	31.0±4.7
Total IQ	96.0±14.9
Verbal comprehension - IQ	100.0±13.9
Perceptual reasoning - IQ	99.0±15.6
Processing speed - IQ	93.0±14.4
Operational memory - IQ	90.0±13.3

GA: gestational age; CPAP: Continuous Positive Airway Pressure; mini-mental: mini-mental state examination; IQ: intelligence quotient.

After medical evaluation, 59.1% PIs presented with clinical school difficulties according to the medical evaluation; 31% scored for lack of attention in the Vanderbilt assessment test, and 35% for hyperactivity. In the psychoeducational evaluation, 38.% presented flaws in the writing test; 57.5% in the reading test; and 42.5% in the mathematical test. In the neuropsychological assessment, 10.9% presented with low or borderline IQ. The other results of the evaluations can be found in Table 2.

**Table 2:** Number of patients whose evaluations were considered altered at school age.

	Normal n (%)	Altered n (%)
Symptom checklist	68 (88.3)	9 (11.7)
Classic mini-mental	27 (32.5)	56 (67.5)
Adapted mini-mental	63 (75.9)	20 (20.1)
ENE - static balance	73 (88.1)	10 (11.9)
ENE - dynamic balance	75 (90.5)	8 (9.5)
ENE - appendicular coordination	68 (82.1)	15 (17.9)
ENE - trunk and limb coordination	69 (83.4)	14 (16.6)
ENE - motor persistence	83 (100.0)	0
ENE - gnosis	52 (61.9)	31 (38.1)
ENE - total	42 (51.2)	41 (48.8)
General neurological examination	69 (83.4)	14 (16.6)
Vanderbilt assessment test - lack of attention*	49 (69.0)	22 (31.0)
Vanderbilt assessment test - hyperactivity*	46 (64.8)	25 (35.2)
Clinical diagnosis of school difficulties	34 (40.9)	49 (59.1)
Piaget cognitive level below expected for the age**	43 (58.9)	30 (41.1)
Writing level**	45 (61.6)	28 (38.4)
Reading level**	31 (42.5)	42 (57.5)
Level of mathematics**	42 (57.5)	31 (42.5)
Total - IQ***	49 (89.1)	5 (10.9)
Verbal comprehension - IQ***	52 (94.5)	2 (5.5)
Perceptual reasoning - IQ***	49 (89.1)	5 (10.9)
Processing speed - IQ***	46 (83.6)	8 (16.4)
Operational memory - IQ***	41 (74.5)	13 (25.5)

Mini-mental: mini-mental state examination; ENE: evolutionary neurological examination by Lefèvre; IQ: intelligence quotient (considered altered when lower than 80) and expressed by the number of children in which it was altered; *only 71 parents answered this questionnaire; **only 73 children underwent this assessment; ***only 54 children underwent this assessment.

When compared to family structure, the group of patients whose family was not structured in the traditional model presented higher frequency (85.0 vs. 50.8%; p=0.006) of clinical school difficulties. Patients with unstructured families obtained higher frequency of flaws in the reading test (78.9 vs. 50.9%; 0=0.029), according to Table 3. In the IQ analysis, there was no difference between groups according to family income and family structure.

**Table 3:** Psychoeducational evaluation and famly structure, gender, gestational age at birth and weight at birth.

	Family structure		
	Non-structured (n=19) n (%)	Traditional (n=53) n (%)	p-value
Piaget cognitive level below the expected for the age	9 (47.4)	21 (39.6)	p=0.372^a^
Difficulty in writing	10 (52.6)	18 (34.0)	p=0.151^b^
Difficulty in reading	15 (78.9)	27 (50.9)	p=0.029^b^
Difficulty in mathematics	10 (52.6)	21 (39.6)	p=0.233^b^
	Gender		
	Female (n=26) n (%)	Male (n=47) n (%)	p-value
Piaget cognitive level below the expected for the age	10 (38.5)	20 (42.5)	p=0.733^b^
Difficulty in writing	8 (30.8)	20 (42.5)	p=0.232^a^
Difficulty in reading	11 (42.3)	31 (66.0)	p=0.043^b^
Difficulty in mathematics	8 (30.8)	23 (48.9)	p=0.104^a^
	Gestational age at birth		
	≤ 30 Weeks (n=36) n (%)	>30 Weeks (n=34) n (%)	p-value
Piaget cognitive level below the expected for the age	10 (45.4)	12 (37.5)	p=0.383^b^
Difficulty in writing	8 (36.7)	12 (37.5)	p=0.581^a^
Difficulty in reading	10 (45.4)	21 (65.6)	p=0.112^b^
Difficulty in mathematics	9 (40.9)	12 (37.5)	p=0.513^a^
	Weight at birth		
	<1500g (n=48) n (%)	≥1500g (n=24) n (%)	p-value
Piaget cognitive level below the expected for the age	21 (43.7)	9 (37.5)	p=0.484^b^
Difficulty in writing	22 (45.8)	6 (25.0)	p=0.723^a^
Difficulty in reading	31 (64.6)	11 (45.8)	p=0.102^b^
Difficulty in mathematics	25 (52.1)	6 (25.0)	p=0.025^a^

^a^Fisher’s exact test; ^b^Pearson’s chi-squared test.

In the analysis regarding maternal schooling, there were no differences in the results of the tests applied at school age.

Boys scored higher in the Vanderbilt Assessment test for lack of attention in comparison to girls (45.0 vs. 12.9%); p=0.003), and presented more frequent flaws in the reading test (66.0 vs. 42.3%; p=0.041), according to Table 3, and in the operational memory IQ test (34.3 vs. 10.0%; p=0.041), according to Table 4.

**Table 4:** Evaluation of the intelligence quotient and family structure, gender, gestational age at birth and weight at birth.

	Family income in minimum wages*		
	<2 (n=15)	≥2 (n=39)	p-value
Total altered - IQ	3 (20.0)	3 (7.7)	p=0.201
Altered verbal comprehension - IQ	2 (13.3)	1 (2.6)	p=0.182
Altered perceptual reasoning - IQ	4 (26.70)	2 (5.13)	p=0.043
Altered processing speed - IQ	3 (20.0)	6 (15.4)	p=0.487
Altered operational memory - IQ	6 (40.0)	8 (20.5)	p=0.137
	Gender		
	Female (n=20)	Male (n=35)	p-value
Total altered - IQ	1 (5.0)	5 (14.3)	p=0.279
Altered verbal comprehension - IQ	1 (5.0)	2 (5.7)	p=0.702
Altered perceptual reasoning - IQ	2 (10.0)	4 (11.4)	p=0.635
Altered processing speed - IQ	2 (10.0)	7 (20.0)	p=0.287
Altered operational memory - IQ	2 (10.0)	12 (34.3)	p=0.043
	Gestational age at birth		
	≤30 Weeks (n=18)	>30 Weeks (n=24)	p-value
Total altered - IQ	3 (16.7)	1 (4.2)	p=0.204
Altered verbal comprehension - IQ	2 (11.1)	0	p=0.176
Altered perceptual reasoning - IQ	3 (16.7)	1 (4.2)	p=0.202
Altered processing speed - IQ	4 (22.2)	3 (12.5)	p=0.336
Altered operational memory - IQ	5 (27.8)	5 (20.8)	p=0.436
	Weight at birth		
	<1500g (n=37)	≥1500g (n=17)	p-value
Total altered - IQ	4 (10.8)	2 (11.8)	p=0.628
Altered verbal comprehension - IQ	2 (5.4)	1 (5.9)	p=0.683
Altered perceptual reasoning - IQ	5 (13.5)	1 (5.9)	p=0.378
Altered processing speed - IQ	8 (21.6)	1 (5.9)	p=0.014
Altered operational memory - IQ	11 (29.7)	3 (17.6)	p=0.277

All variables are expressed in n (%), Fisher’s exact test; *family income defined in minimum wages; IQ: intelligence quotient (considered altered when ≤79).

At the evaluation of gestational age, PIs with GA<30 weeks using the ultrasound presented higher frequencies of flaws in the adapted mini-mental test (32.5 vs. 10.3%; p=0.035). In the comparison regarding GA, there was no difference in the results of the psychoeducational or neuropsychological evaluations.

Regarding the Apgar score at 1 and 5 minutes after birth and type of birth, there were no differences regarding neurological, academic or IQ tests. We considered the tests to be altered when Apgar score was<7. The PIs whose weight at birth was <1500g presented with higher frequency of change in the mathematical tests (52.1 *vs* 25.0%; p=0.029) and processing speed (21.6 *vs* 5.9%; p = 0.012), according to Table 4.

The PIs with early sepsis had higher frequency of change in the Vanderbilt Assessment test for lack of attention (36.5 *vs* 11.8%; p=0.041) and hyperactivity (42.3 *vs* 12.0%; p=0.042). For PIs with late sepsis, more changes appeared in the diagnosis of school difficulties made by the child neurologist (71.4 *vs* 50.0%; p=0.041). There were no differences when the assessment involved neuropsychological and psychoeducational tests.

There were no differences in the frequency of changes in assessment tests at school age when compared to the presence of the variables: retinopathy of prematurity (ROP), ophthalmological changes and days in invasive mechanic ventilation.

When the presence of HPIV was assessed alone, there was no difference between those with or without HPIV in the results of the evaluations. However, among those who had HPIV, the ones with grades III and IV hemorrhage presented higher levels of change in the Piaget operational stage (83.3 *vs* 35.3%; p=0.013).

In the multiple regression analysis, there was a relationship between GA, birth and score in the classic mini-mental test (p=0.04), total IQ (p=0.047), perceptual reasoning IQ (p=0.035) and processing speed IQ (p=0.036). There was also a relationship between weight at birth and classic (p=0.004) and adapted mini-mental (p=0.007) tests; between time of invasive mechanic ventilation and classic mini-mental (p=0.049); and between lower maternal age and processing speed IQ (p=0.033). There was no relation between the other assessed variables.

## DISCUSSION

This study assessed the neurological, cognitive and academic path of students who were born premature and had no severe sequelae. In this context, we expected a low incidence of major disabilities and sequelae, such as deafness, blindness, cerebral palsy and intellectual disability. However, students who were born premature presented with high levels of insufficient academic performance in reading, writing and mathematics.

Our results are in accordance with the findings in the literature. In Brazil, Riechi et al., in 2011, found worse academic performance of PIs when compared to students who were born at term.^17^ In Australia, Taylor et al*.*, in a study including 194 PIs with GA <30 weeks, showed poorer performance in standardized reading (22%), writing (20%) and mathematical (40%) tests at the age of 7 when compared with controls born at term (7, 6 and 11%, respectively).^20^

The levels of flaw in cognitive evaluations, and especially in school-related assessments, were considerable. One of the influencers for the performance to be worse than expected was the low family income of the studied sample, once poverty and social risk play an important role in the cognitive development of children.^33^ There was no association between lower family income, lower maternal schooling and poor performance in WISC or in the psychoeducational evaluations. Likewise, this fact can be explained by the homogeneity of the sample, in which family income and maternal schooling were low, and factors such as social status and maternal schooling have an impact both on cognitive development and biological factors.^34^ Therefore, with more access to health services and better socioeconomic status, in developed countries, in the past few years, the cognitive development of premature infants has tended to improve,^35^ which we could not observe in this cross-sectional sample.

Boys presented higher frequency of flaw in reading tests, operational memory IQ and lack of attention, which is compatible with the general prevalence of these disabilities, much more common in the male gender.^36^ ^,^ ^37^ ^,^ ^38^

The lowest GA (<30 weeks) was associated with the cognitive changes suggested by the adapted mini-mental test, but not suggested by the IQ tests. This finding can be explained by the fact that the mini-mental is a screening test, less specific than WISC. The diagnosis of an intellectual disability, despite being often and objectively based on the result of the IQ test, by definition occurs due to an adaptive difficulty of patients regarding the challenges imposed by the environment.^39^ It is possible that a more sensitive and less specific screening test, such as the mini-mental, could be less sensitive to detect such a change. Likewise, lower GA was associated with more changes in the neurological examination. Premature infants, even in the absence of cerebral palsy, present with higher levels of change in the neurological examination.^40^

There was no association between neonatal asphyxia, suggested by the Apgar score, and cognitive and school-related changes. Another study of similar design showed that perinatal asphyxia was associated to lower IQ levels at school age.^41^ This study considered the Apgar score below 7 as altered, thus also considering the cases of mild hypoxia, once the objective was to assess the survivors who did not have severe sequelae, whereas other studies considered the score lower than 3 (severe hypoxia).^42^ Even though a series of factors limit the use of the Apgar score as a prognostic factor, especially due to the little homogeneity of its application by the several professionals, it is still indicated for the long-term evaluation of development as a major risk factor, especially considering the 5^th^ minute after birth.^43^

Lower maternal age was associated with lower rates of processing speed IQ, which is connected both to symptoms of lack of attention of ADHD and those referring to impulsivity and behavior. Lower maternal age in premature infants is associated with increased risk of behavioral issues for the child.^44^

Early neonatal sepsis was associated with the diagnostic suggestion of ADHD, both the lack of attention and the hyperactive types, without, however, being associated with other aspects of cognitive and academic development. In a French cohort of PIs, there was no association between neonatal sepsis (early or late) and ID at the age of 5.^44^ However, a multicenter analysis showed higher risk of cognitive impairment in EPIs who had late bacteremia.^8^ The inflammatory mediators produced in sepsis can be related with changes in neural connections that act as a neurobiological base for ADHD.

No association was found between prolonged time of invasive mechanic ventilation and changes in cognitive and academic development. However, the prolonged use of inhaled oxygen or the presence of a clinical diagnosis of bronchopulmonary dysplasia, which is considered as a major risk factor for school difficulties, were not analyzed.^45^

The presence of HPIV alone was not an independent risk factor for cognitive delay. The presence of severe HPIV was associated with changes in the Piaget operational state, which demonstrates changes in the cognitive learning process of PIs. Extensive brain injury has a robust impact factor on cognitive development and difficulties in reading, writing and mathematics.^46^ Likewise, it is possible to point out that mild HPIV (grades I and II) do not have an influence on school and cognitive development.^47^ Abnormalities found in magnetic resonance images of PIs, especially in the deep grey matter, can be associated with memory damage, operational memory and learning at school age.^48^ Magnetic resonance imaging studies associated volumetric reduction and changes present in the white matter, deep grey matter (thalamus) and cerebellum with poor performance in cognitive evaluations in adolescence.^48^

There was a major prevalence of school difficulties in the studied population. The development of school difficulties, especially that of reading and writing disorders, is usually associated with delayed language acquisition, and PIs often present with atypical language development, with higher incidence of delayed acquisition of expressive language.^49^

Study limitations were reduced sample size, when compared to similar studies; absence of a control group with students born at term; difficulty to conclude the evaluation of children; and difficulty to look for some data from the neonatal period in the records.

Despite these limitations, it was possible to define an academic and cognitive profile of PIs that is more adequate to the Brazilian cultural reality, showing major impact of prematurity in the cognitive and school-related development of the survivors, even if these are free of more severe sequelae that can be early identified (such as cerebral palsy and severe developmental limitations).

With these results, it is recommended that the follow-up of PIs in risk outpatient clinics, especially for EPIs, be continued until after the children begin their school life, and that education and psychology professionals be included in the multidisciplinary staff that conduct this follow-up.
